# Integrating proteomic and phosphoproteomic data for pathway analysis in breast cancer

**DOI:** 10.1186/s12918-018-0646-y

**Published:** 2018-12-21

**Authors:** Jie Ren, Bo Wang, Jing Li

**Affiliations:** Department of Bioinformatics and Biostatistics, School of Life Sciences and Biotechnology, Shanghai Jiao Tong University, Shanghai, 200240 China

**Keywords:** Proteomics, Phosphoproteomics, Integration, Pathway analysis, Breast cancer

## Abstract

**Background:**

As protein is the basic unit of cell function and biological pathway, shotgun proteomics, the large-scale analysis of proteins, is contributing greatly to our understanding of disease mechanisms. Proteomics study could detect the changes of both protein expression and modification. With the releases of large-scale cancer proteome studies, how to integrate acquired proteomic and phosphoproteomic data in more comprehensive pathway analysis becomes implemented, but remains challenging. Integrative pathway analysis at proteome level provides a systematic insight into the signaling network adaptations in the development of cancer.

**Results:**

Here we integrated proteomic and phosphoproteomic data to perform pathway prioritization in breast cancer. We manually collected and curated breast cancer well-known related pathways from the literature as target pathways (TPs) or positive control in method evaluation. Three different strategies including Hypergeometric test based over-representation analysis, Kolmogorov-Smirnov (K-S) test based gene set analysis and topology-based pathway analysis, were applied and evaluated in integrating protein expression and phosphorylation. In comparison, we also assessed the ranking performance of the strategy using information of protein expression or protein phosphorylation individually. Target pathways were ranked more top with the data integration than using the information from proteomic or phosphoproteomic data individually. In the comparisons of pathway analysis strategies, topology-based method outperformed than the others. The subtypes of breast cancer, which consist of Luminal A, Luminal B, Basal and HER2-enriched, vary greatly in prognosis and require distinct treatment. Therefore we applied topology-based pathway analysis with integrating protein expression and phosphorylation profiles on four subtypes of breast cancer. The results showed that TPs were enriched in all subtypes but their ranks were significantly different among the subtypes. For instance, p53 pathway ranked top in the Basal-like breast cancer subtype, but not in HER2-enriched type. The rank of Focal adhesion pathway was more top in HER2- subtypes than in HER2+ subtypes. The results were consistent with some previous researches.

**Conclusions:**

The results demonstrate that the network topology-based method is more powerful by integrating proteomic and phosphoproteomic in pathway analysis of proteomics study. This integrative strategy can also be used to rank the specific pathways for the disease subtypes.

**Electronic supplementary material:**

The online version of this article (10.1186/s12918-018-0646-y) contains supplementary material, which is available to authorized users.

## Background

Following the quick accumulation of large-scale genome, transcriptome and other omics data, some studies or approaches integrating multiple omics data into pathway analysis have been reported [1–4]. Mass-spectrometry-based proteomics provides insights into cell-type protein expression patterns, post-translational modifications (PTMs) and protein–protein interactions [5–7]. As the most common PTMs, up to 30% of all human proteins may be modified by kinase activity (Phosphorylation), and kinases are known to regulate the majority of cellular signal pathways. To date, how to integrate the information of protein expression, PTMs and protein interactions in pathway analysis is still a big challenge.

Signal pathways describe a group of molecular in a cell that work together to control one or more cell functions, such as cell division or cell death. Pathway analysis gives an insight into the underlying mechanism in a given condition and makes it more explanatory in comparison with the studies at individual gene or protein level. Pathway analysis methods include gene set analysis and topology-based analysis. Gene set methods only consider the set of genes/proteins in the pathways while the topology-based methods use both genes/proteins and the interactions among them. Gene set methods consist of Over-Representation Analysis (ORA) based on the Hypergeometric test or Fisher exact test [8, 9] and Functional Class Score (FCS) based on ranked gene list and Kolmogorov-Smirnov (K-S) test [10]. The ORA only considers the differentially-expressed (DE) genes and the representative tools of ORA include DAVID [11], Onto-Expression [9], GenMAPP [12], GOMiner [13], GOstat [14] and so on. FCS considers the position of all genes in the ranked list, which is produced by a selected statistical test for differential expression, such as Gene Set Enrichment Analysis (GSEA) [15], Gene Set Analysis (GSA) [16] and so on. Topology-based pathway analysis integrate both changes in expression level and in topology of protein/gene interaction network, which includes Signal pathway impact analysis (SPIA) [17] and Bayesian Pathway Analysis (BPA) [18]. In SPIA, the score of the pathway is based on the impact analysis consisting of two types of evidence. One is the over-representation of DE genes in a given pathway and the other is the abnormal perturbation of that pathway, which is measured by propagating expression changes across the pathway topology.

In this work, we tried to integrate proteomic and phosphoproteomic data in pathway analysis in breast cancer and its subtypes. The results showed that integrating protein and phosphorylation differential expression with the network-topology based method can identify the target pathways more accurately. What’s more, we also identified the top ranked pathways in four subtypes of breast cancer specifically.

## Methods

### Proteomics data and preprocessing

The proteomic and phosphoproteomic data of breast cancer in this study included 77 tumor samples and 3 normal breast tissue samples, which were downloaded from Clinical Proteomic Tumor Analysis Consortium (CPTAC). The process of quality control and normalization for both the proteomic and phosphoproteomic data was presented in Mertin et al.’s work [5]. As the result, 12,553 proteins (10,062 genes) and 33,239 phosphosites with their relative abundances quantified across tumors were used in this work. The missing value in the data matrix was filled with the minimum value.

### Integrating proteomic and phosphoproteomic data

Since ORA, GSEA and SPIA are the representatives of three kinds of pathway analysis, which are Over-Representation analysis, Functional Class Score and topology-based pathway analysis, we used these three strategies to do pathway analysis. We used R package ‘HTSanalyzeR’ [19] to do ORA, GSEA pathway analysis and another R package ‘SPIA’ [17] to do SPIA pathway analysis. *P*-values for pathway analysis resulting from the permutation (*n* = 2000) were provided in Additional File 1: Table S1.

Different methods of pathway analysis require different input data. For ORA, the input file is the list of DE proteins/modifications or the intersection of the DE protein and phosphoprotein as an integration (Student’s t-test, with BH-adjusted *p* < 0.05). The input file for GSEA method in our study was the list of all proteins/phosphoproteins with fold change between the case and control. We summed up and sorted the fold changes for the overlapping proteins in the protein expression and phosphorylation profiles as the integrated information for GSEA. As for SPIA, the input files consisted of the topology of the pathways downloaded from KEGG database and the DE proteins with their fold change. The topology changes of the pathways could be calculated by the ‘SPIA’ R package. The input for SPIA was the intersection list of the DE proteins and DE phosphoproteins with the sum of their fold change.

### Performance evaluation

For the performance evaluation of pathway analysis, a widely used validation method is using the ranks of the target pathways in disease that have been validated or curated in publication, topper rank is better. This method is proposed in PADOG [20] and used in other studies of pathway analysis methods comparison [21, 22].

We manually selected twelve breast cancer related TPs from literatures. Most of TPs are mentioned in the work about comprehensive molecular portraits of human breast tumors [23, 24] and the others are also widely accepted. The TPs and their references were listed in Table 1.

**Table 1:** The target pathways for breast cancer

KEGG ID	Pathway name	Reference
hsa04014	Ras signaling pathway	[24]
hsa04151	PI3K-Akt signaling pathway	[23]
hsa04010	MAPK signaling pathway	[23]
hsa04150	mTOR signaling pathway	[45, 47, 49]
hsa04310	Wnt signaling pathway	[23]
hsa04115	p53 signaling pathway	[23, 33]
hsa01521	EGFR tyrosine kinase inhibitor resistance	[23]
hsa04012	ErbB signaling pathway	[23]
hsa04510	Focal adhesion	[34, 35]
hsa04350	TGF-beta signaling pathway	[60]
hsa04110	Cell cycle	[24]
hsa05200	Pathways in cancer	[23]

## Results

**Fig. 1:**
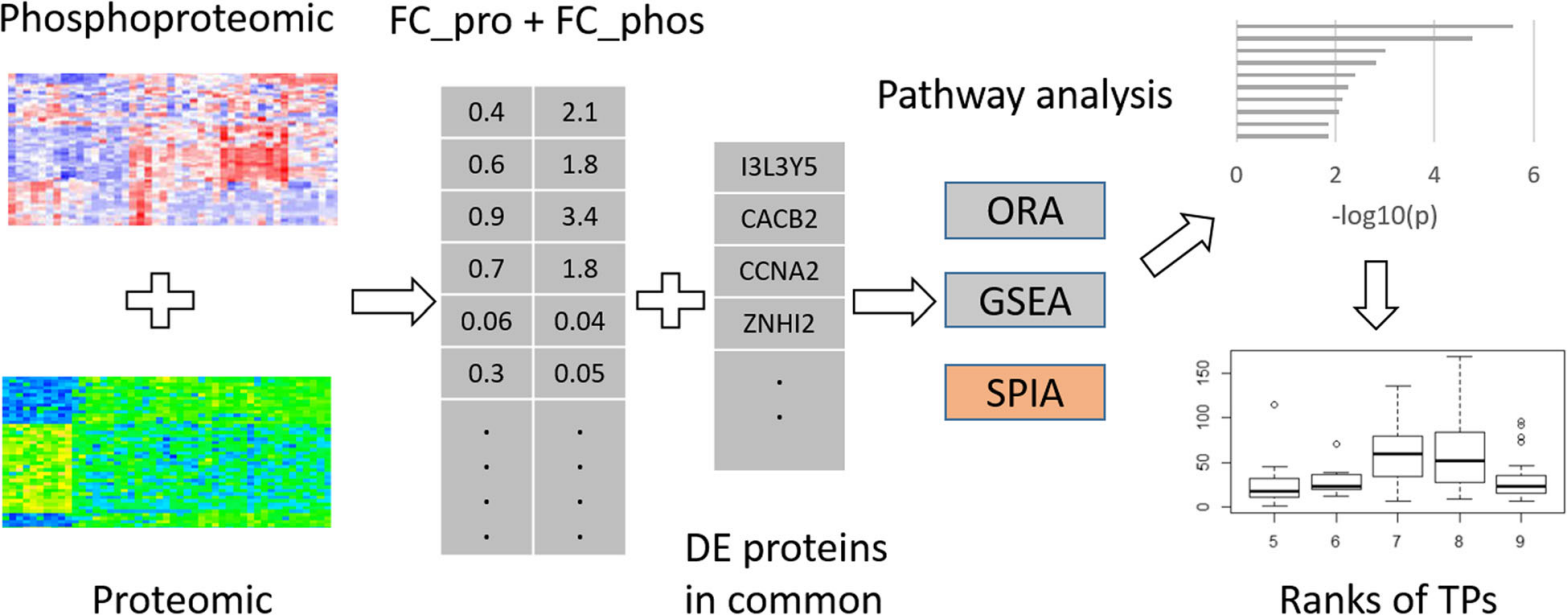
A workflow of integrating proteomic and phosphoproteomic data in pathway analysis. Firstly, the fold change of the two protein lists from proteomic and phosphoproteomic respectively were summed up. The interaction of the DE proteins and the DE phosphoproteins were also recorded. Secondly, we performed ORA, GSEA and SPIA pathway analysis by using the integrated information, and obtained the ranks of the target pathways. Finally, the methods were evaluated by the ranks of the target pathways

The workflow of integrating proteomic and phosphoproteomic data to perform pathway analysis was shown in Fig. 1. Firstly, integrating information from proteomic and phosphoproteomic data were used as the input of pathway analysis. Then, we processed ORA, GSEA and SPIA pathway analysis on the integrated information. Finally, the methods were evaluated by the ranks of the TPs. In our study, we identified 2337 DE proteins and 3973 DE phosphoproteins respectively. The intersection of the two lists were 641 proteins.

### Performance evaluation of pathway analysis with protein expression and/or phosphorylation profiles

**Fig. 2:**
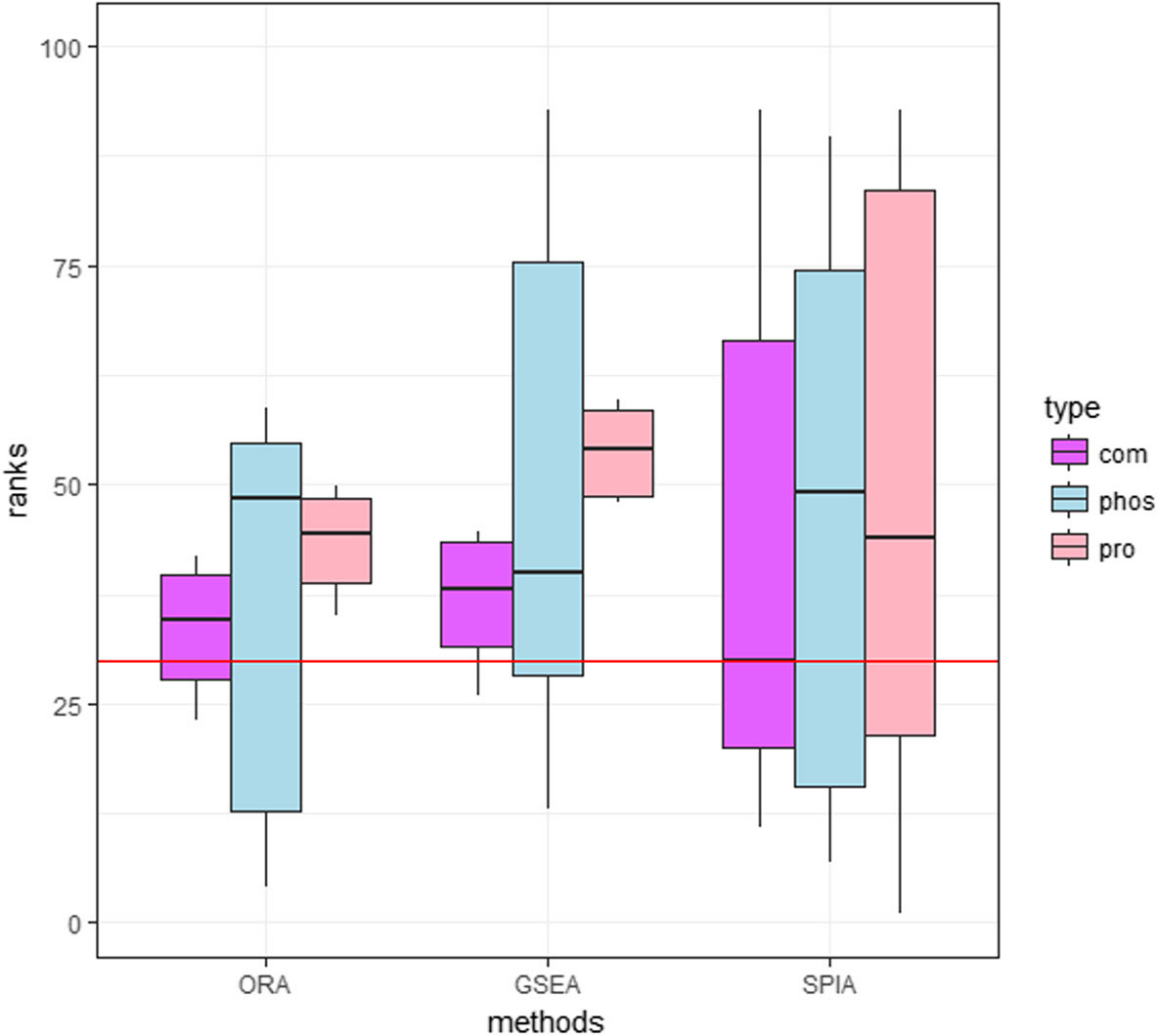
Box plot of the ranks for the target pathways in breast cancer. The ranks were normalized in the range of 1 to 100. Lower rank is, better performance of the method is. The orchid, blue and pink represent the method based on the integrated information, information from proteomic and phosphoproteomic data

To assess the integrating strategies in pathway ranking with proteomics data, we compared the ranks of TPs in three kinds of pathway analysis methods with integrated information, including protein expression and phosphorylation datasets separately. Fig. 2 showed the box plots of normalized ranks in the range of 1 to 100 (the lower, the better). It could be concluded from the figure that all the pathway analysis methods performed better using the integrated data than using single information. Especially, topology-based pathway strategy introduced in SPIA performed best as the median rank of rankings for all the TPs was lower than any other methods.

Besides the TPs, we found nineteen pathways appearing in the overlap of the top 50 pathway ranking lists of three kinds of pathway analysis methods with integrated information, such as Fanconi anemia pathway, GABAergic synapse (as shown in Table 2). Although these pathways are not validated, as well as TPs to be related to breast cancer, there are still researches indicated the correlation with breast cancer. For example, Fanconi anemia pathway is closed linked to breast and ovarian cancer susceptibility gene BRCA1 [25, 26]. Abnormal GABA expression or GABAergic participation has been described in primary colon, gastric, ovarian, pancreatic, and breast cancers [27], while GABA and GABAergic participation are involved in GABAergic synapse [28]. What’s more, it has been reported that morphine can stimulate angiogenesis by activating proangiogenic and survival-promoting signaling and promote breast tumor growth [29].

**Table 2:** The overlap of top 50 ranking pathways in three methods with integrated information

KEGG ID	Pathway name
hsa03460	Fanconi anemia pathway
hsa04020	Calcium signaling pathway
hsa04024	cAMP signaling pathway
hsao4060	Cytokine-cytokine receptor interaction
hsa04261	Adrenergic signaling in cardiomyocytes
hsa04340	Hedgehog signaling pathway
hsa04710	Circadian rhythm
hsa04713	Circadian entrainment
hsa04723	Retrograde endocannabinoid signaling
hsa04724	Glutamatergic synapse
hsa04725	Cholinergic synapse
hsa04727	GABAergic synapse
hsa04912	GnRH signaling pathway
hsa04914	Progesterone-mediated oocyte maturation
hsa05020	Prion diseases
hsa05032	Morphine addiction
hsa05166	HTLV-I infection
hsa05216	Thyroid cancer
hsa05217	Basal cell carcinoma

### Pathway rankings in subtypes of breast cancer

**Fig. 3:**
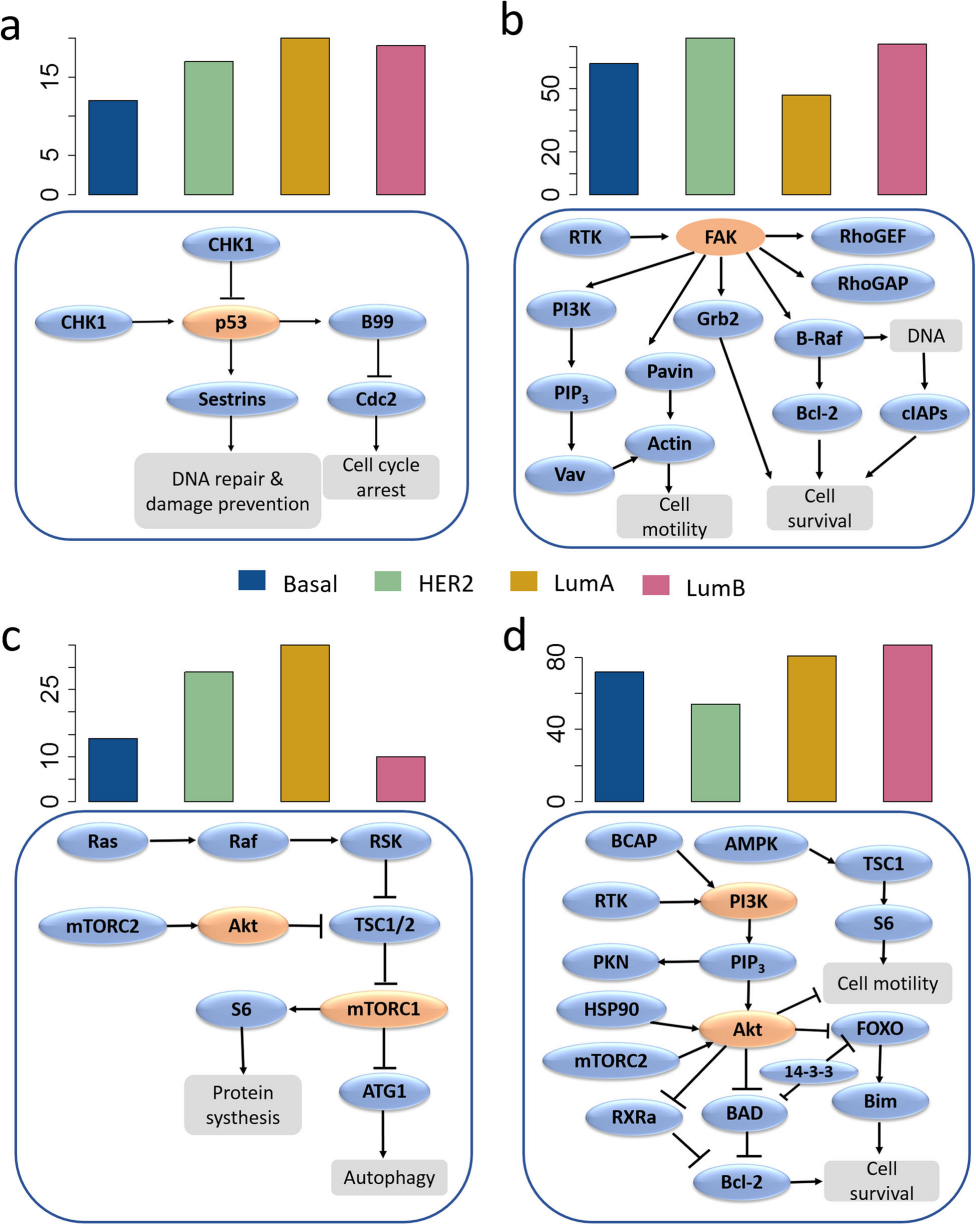
Comparison of four TPs in breast cancer subtypes. The ranks of p53 signaling pathway (**a**), Focal adhesion (**b**), mTOR signaling pathway (**c**) and PI3K-Akt signaling pathway (**d**). The bar plot above describes the ranks of the TP in four subtypes and the below one shows the changed molecular and the hot member of the pathway

The subtypes of breast cancer, which consist of Luminal A, Luminal B, Basal and HER2-enriched [23, 30], are various in prognosis and require distinct treatment [24, 31]. Genomic, transcriptomic, and proteomic analyses of the breast cancer also reveal subtypes would differ in pathway activity [32]. If the specific pathways and the underlying mechanism of each subtype are identified, more precision treatments can be applied. Based on the performance evaluation of different pathway analysis, we analyzed and ranked the perturbed pathways for each subtype by integrating protein expression and modification profiles using the network-topology based approach. The results showed ranking of the perturbed TPs in four subtypes (Additional File 2: Figure S1). Some pathways, like cell cycle and pathway in cancer, were among top10 rankings in all of subtypes. The ranks of other TPs were different among the subtypes though they all play important roles in four subtypes. We selected representative top-ranked pathways in each subtype and display them in Fig. 3.

As shown in Fig. 3a, p53 pathway ranked lowest in the Basal-like breast cancer type and ranked lower in Luminal A than in Luminal B. It is reported that TP53 are the most recurrently mutated genes in breast cancer, with frequency of 84% in Basal-like tumors [23] and p53 pathway remains largely intact in Luminal A cancers but is often inactivated in the more aggressive Luminal B cancers [33].

In accordance with previous research, expression levels of Focal adhesion kinase (FAK/PTK2) are correlated strongly with poor tumor differentiation and significantly associated with HER2 overexpression in breast cancer [34]. The highest level of FAK (Y861) and the lowest level of epidermal growth factor receptor 2 (HER2) activity can be observed in MDA-361 cells (ER+/HER2+ cell) [35]. As FAK is the important role in the Focal adhesion pathway, we can infer that the activation of the Focal adhesion pathway was negative correlated with the expression of HER2. The rank of Focal adhesion pathway was lower in HER2- subtypes (Luminal A and HER2) than HER+ subtypes (Luminal B and Basal), as shown in Fig. 3b.

PI3K/AKT/mTOR pathway is a key intracellular signaling system that drives cellular growth and survival. Hyperactivation of this pathway is implicated in the tumorigenesis of ER+ breast cancer [36–45]. Besides, the pathway is also important in Triple-negative breast cancer [46] and HER2-overexpressing breast cancer [47]. Preclinical studies indicate that inhibitors of the pathway can act synergistically with trastuzumab in resistant cells [48].

Many studies have established that mTOR pathway has tightly interaction with PI3K-AKT and MAPK signaling pathways. Inhibition of mTORC1, an important part of mTOR pathway, leads to MAPK pathway activation through a PI3K-dependent feedback in human cancer [49]. It can be verified by the ranks of these pathways in four breast subtypes, the low rank of mTOR pathway corresponded to the high rank of PI3K-Akt signaling pathway (Fig. 3c and d). Luminal-type cells might use the MEK-ERK pathway to a lesser extent and seem to be more dependent on the PI3K pathway, shown by the preferential occurrence of PI3K mutations in this subtype [10]. As show in Fig. 3d, PI3K-Akt signaling pathway in Luminal subtype ranked higher than the other two subtypes.

**Fig. 4:**
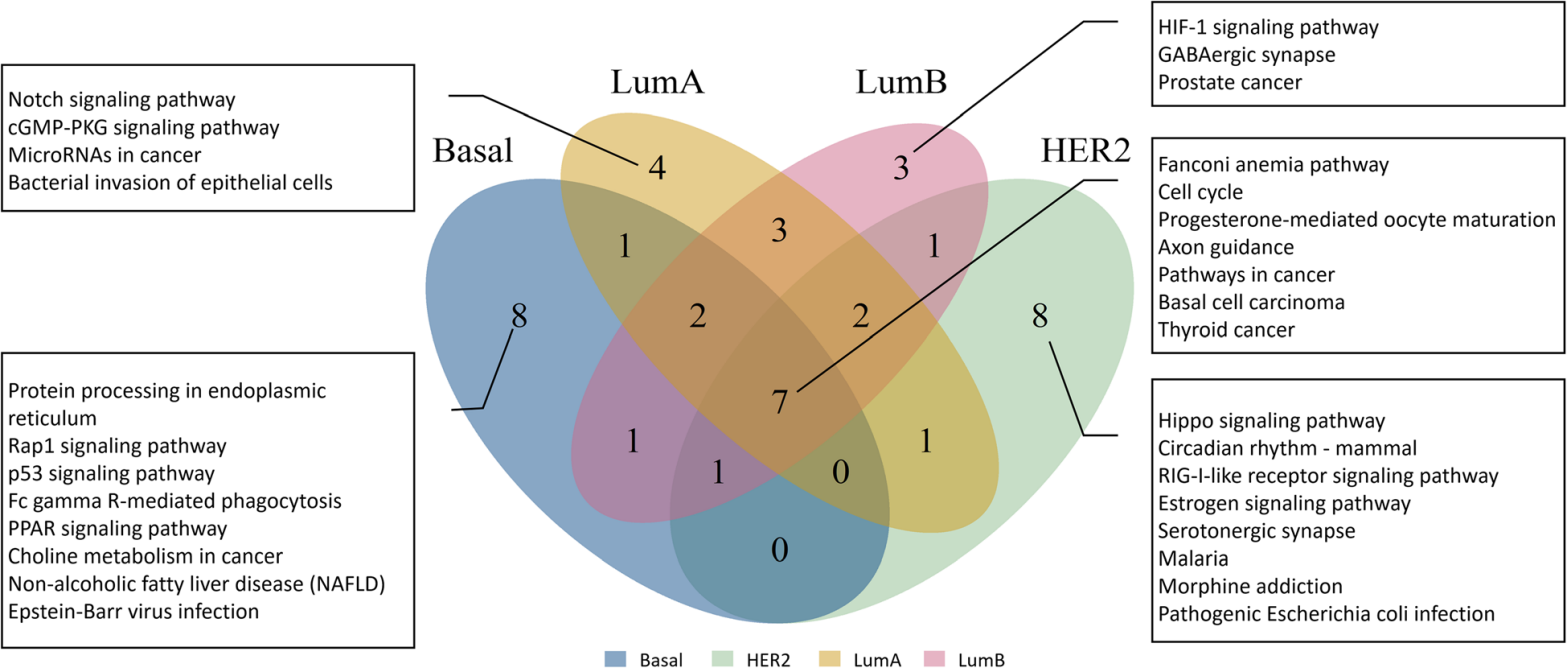
The Venn diagram of top 20 ranking pathways for four subtypes. The top 20 pathways were ranked by the SPIA method with integrated information

We also took a look at the top 20 ranked pathways for each subtype of breast cancer. There were 7 common pathways among the four subtypes. Besides two TPs cell cycle and pathways in cancer, the other common pathways have been reported to be related with breast cancer pathways which consist of Fanconi anemia pathway [50], Progesterone-mediated oocyte maturation [51, 52], Axon guidance [53], Basal cell carcinoma [54] and Thyroid cancer [55, 56]. As shown in Fig. 4, some pathways were specifically ranked in top 20 for Basal, HER2, Luminal A and Luminal B respectively. This result indicated that the subtypes share some common molecular mechanisms during carcinogenesis and development, but the differences between them also exist. For example, as we mentioned above, p53 pathway is significantly perturbed in Basal-like subtype but it also play key role in the other three subtypes [23, 57]. Notch pathway in Luminal breast cancer is activated more than in Basal and HER2 subtypes [58, 59].

## Discussion

Expression and modification describe the in vivo changes of proteins in cancer proteome at different views. The pathway analysis based on the information at single level, such as protein expression or protein phosphorylation alone, often brings high risk of both false positive and false negative due to technological limitations. To the best of our knowledge, the integration proteomic and phosphoproteomic data in pathway analysis in cancer has not been evaluated and reported. In this study, the pathway analysis was performed and compared using the integration of proteomic and phosphoproteomic data in CPTAC’s breast cancer dataset. Moreover we tried to find the different patterns in pathway ranking among the subtypes.

Our results suggested that both differential expression of proteins and phosphorylation were useful for identifying the important pathways in cancer or cancer subtypes. Furthermore, the integration of protein expression and modification profiles could provide more comprehensive information and rank TPs more accurately. Although the ranking lists of three kinds of pathway analysis were different, some consistent results were observed since the expression change of proteins and phosphoproteins are used in all of strategies. While the GSEA requires the fold change of all proteins, it has more complete information reflecting the expression profile. SPIA needs the topology information of the pathways in addition, which can provide detailed influence between the nodes of pathways.

We also tested the performance using the union of DE proteins and phosphoproteins information in pathway ranking, but poor accuracy was obtained. It’s possibly because of too much noise in individual omics data. In order to control the risk of false positive, the intersection of the DE proteins or DE PTMs were used as input in this study that might be too conservative. Because only one dataset was tested here, for some new pathways in top ranking list, more independent proteomics datasets in cancer need to be processed and validated in the future.

## Conclusions

Integrative pathway analysis by combing the information from protein expression, protein modification and the topology of protein interaction network is more efficient way to identify key pathway in breast cancer. Pathway ranking in certain subgroup of patients can provide insight into the specific mechanisms and be helpful for the precision medicine for each subtype.

## Additional files

Additional file 1: **Table S1.** The results of different pathway ranking methods. (XLSX 89 kb)

Additional file 2: **Figure S1.** Ranks of TPs in subtypes of breast cancer. (DOCX 17 kb)

## Abbreviations

BPA: Bayesian Pathway Analysis
CPTAC: Clinical Proteomic Tumor Analysis Consortium
DE: Differentially expressed
FAK: Focal adhesion kinase
FCS: Functional Class Score
GSA: Gene Set Analysis
GSEA: Gene Set Enrichment Analysis
K-S: Kolmogorov-Smirnov
ORA: Over-Representation Analysis
PTMs: Post-translational modifications
SPIA: Signal pathway impact analysis
TPs: Target pathways
