# Supplementary material for: Integrating proteomic and phosphoproteomic data for pathway analysis in breast cancer
Source: BMC Syst Biol. 2018 Dec 21;12(Suppl 8):130. doi: 10.1186/s12918-018-0646-y (PMC6302460; doi:10.1186/s12918-018-0646-y)
Supplement: Supplementary file 2 — Figure S1. Ranks of TPs in subtypes of breast cancer. (DOCX 17 kb) [file 12918_2018_646_MOESM2_ESM.docx]

**Additional file 1: Figure S1.** Rankings of TPs in subtypes of breast cancer.

**Additional file 1: Figure S1**
